# Hemispheric Asymmetry in Cortical Auditory Processing: The Interactive Effects of Attention and Background Noise

**DOI:** 10.3390/audiolres16010017

**Published:** 2026-01-28

**Authors:** Anoop Basavanahalli Jagadeesh, Ajith Kumar Uppunda

**Affiliations:** 1 School of Speech, Language, Hearing, and Occupational Sciences, University of Montana, Missoula, MT 59812, USA; 2 Department of Audiology, All India Institute of Speech and Hearing, Mysuru 570006, India; ajithkumar18@gmail.com

**Keywords:** cortical auditory evoked potentials (CAEPs), attention, hemispheric asymmetry, speech perception, auditory processing

## Abstract

**Background/Objectives**: Speech processing engages both hemispheres of the brain but exhibits a degree of hemispheric asymmetry. This asymmetry, however, is not fixed and can be shaped by stimulus-related and listener-related factors. The present study examined how background noise and attention influence hemispheric differences in speech processing using high-density cortical auditory evoked potentials (CAEPs). **Methods**: Twenty-five young adults with clinically normal hearing listened to meaningful bisyllabic Kannada words under two background conditions (quiet, speech-shaped noise) and two attentional conditions (active, passive). N1 peak amplitudes were compared between the left and right hemispheres across conditions using linear mixed-effects modeling. **Results**: Results revealed significantly larger N1 amplitudes in the left hemisphere and during active compared to passive listening, confirming left-hemisphere dominance for speech processing and robust attentional modulation. In contrast, background noise did not significantly modulate N1 amplitude or hemispheric asymmetry. Importantly, a significant Hemisphere × Attention interaction indicated that hemispheric asymmetry depended on attentional state, with clear left-hemisphere dominance being observed during active listening in both quiet and noise conditions, whereas hemispheric differences were reduced or absent during passive listening, irrespective of background. **Conclusions**: Together, these findings demonstrate that attentional engagement, rather than background noise, plays a critical role in modulating hemispheric specialization during early cortical speech processing, highlighting the adaptive nature of auditory cortical mechanisms in challenging listening environments.

## 1. Introduction

Speech processing in the human brain engages both cerebral hemispheres [1]. Yet, there exists an asymmetry in the way each hemisphere contributes to processing different aspects of speech. The left hemisphere is predominantly involved in the processing of segmental features of speech, such as phonetic, syntactic, and semantic structures, which require fine-grained temporal resolution. In contrast, the right hemisphere plays a dominant role in processing suprasegmental features of speech, including prosody, intonation, and melodic elements, and even music [2,3,4]. For example, complex stimuli like speech with its rapid spectro-temporal changes are shown to elicit stronger cortical activation in the left hemisphere’s auditory cortex [5,6]. However, the degree of hemispheric asymmetry in auditory processing is not static, and both stimulus-related factors, such as stimulus spectral composition, intensity, presence of background noise, etc., as well as listener-related factors such as hearing acuity, age, handedness, etc., can influence the extent of cerebral asymmetry in auditory processing [7,8].

One way to explore these hemispheric differences in auditory processing is through Cortical Auditory Evoked Potentials (CAEPs), which are well-established neural markers of cortical auditory function. CAEPs exhibit hemispheric asymmetries, with studies consistently revealing larger response amplitudes (e.g., N1 peak) over the left hemisphere compared to the right, particularly for speech stimuli [7,9]. Additionally, some studies suggest that N1 latency can also be earlier in the left hemisphere, possibly reflecting its greater sensitivity to rapid temporal changes in speech [10,11].

Beyond their role as neural markers of auditory encoding, CAEPs are influenced by both bottom-up (stimulus-driven) and top-down (cognitive) mechanisms, both of which can interact with hemispheric specialization. While bottom-up factors, such as stimulus spectral composition and intensity [12], directly affect the robustness of CAEP responses, top-down processes like selective attention can enhance neural responses to relevant stimuli [13,14]. This makes CAEPs particularly useful in understanding how cognitive factors interact with neural auditory processing. Given the interplay between sensory and cognitive processes in speech perception, investigating how both background noise and attention interact to shape hemispheric asymmetry in CAEPs provides a valuable framework for understanding auditory cortical plasticity in challenging listening environments.

Studies have consistently shown that background noise influences the cortical representation of speech [11,15,16]. Generally, the presence of background noise leads to CAEP response amplitudes being smaller and latencies being delayed [17,18]. Of particular relevance to the current study is the finding that background noise modulates the cerebral asymmetry typically observed in the cortical representation of speech [19,20]. Specifically, using magnetic mismatch negativity (MMNm), Shtyrov et al. (1998) [20] demonstrated that while speech stimuli in silence elicited stronger responses in the left auditory cortex, introducing background white noise significantly altered this asymmetry. Shtyrov et al. (1999) [19] further expanded on this finding and showed that a similar shift towards the right hemisphere in the presence of background noise was observed even in the obligatory (P1-N1-P2 components) CAEP responses. This suggests that background noise diminishes the predominance of the left hemisphere, possibly reflecting recruitment of additional auditory processing networks to maintain speech perception under challenging conditions.

Similarly to background noise, attention is also observed to modulate the representation of speech in the brain. In auditory neuroimaging studies, ‘attention’ is operationally defined as active listening to task-relevant sounds, in contrast to passive listening. Using an event-related fMRI auditory oddball task, Stevens et al. (2005) [21] demonstrated greater activation of the right hemisphere, compared to the left, in response to the detection of task-relevant low-probability stimuli (oddballs), irrespective of the participants’ age or gender. Petit et al., (2007) [22] revealed that there exists a preferential engagement of the right hemisphere in auditory processing while interacting with attention. Alexander et al. (1996) [23] recorded auditory P300 responses to auditory oddball stimuli, and showed that the P300 amplitudes were significantly larger in the right hemisphere compared to the left hemisphere. In contrast, Poeppel et al. (1996) [24] demonstrated that the M100 responses (the MEG analog of ERP N100 or N1) were comparable between the two hemispheres under a passive attention condition, while an active discrimination task resulted in larger M100 responses over the left hemisphere.

Taken together, these findings suggest that attention dynamically modulates hemispheric asymmetry in auditory processing, with a general trend toward right hemisphere dominance under active attention. However, this pattern is not unequivocal. In contrast, background noise has been shown to shift auditory processing toward the left hemisphere. Despite these opposing trends, previous research has largely treated attention and background noise as separate modulators. As a result, their combined influence on hemispheric asymmetry remains underexplored. Given these differing lateralization patterns, the present study aims to investigate whether attention and background noise interact to influence cortical auditory evoked potentials (CAEPs) for speech stimuli. Specifically, we examine whether each factor exerts an independent effect on lateralization or whether their interaction reveals a more dynamic modulation of cortical engagement under complex listening conditions.

## 2. Materials and Methods

### 2.1. Participants

A total of 25 participants (10 females, 15 males) volunteered for this study. All participants were young adults who were native speakers of the Kannada language and in the age range of 18–35 years (Mean age = 23.5 years) with pure-tone thresholds in the audiometric frequencies between 250 Hz and 8000 Hz being less than 15 dB HL. Because handedness can strongly influence hemispheric specialization [25], we ensured that all participants were right-handed using the Edinburgh Handedness Inventory [26]. Further, all participants were ensured to have no current or past history of any neurological, neuropsychiatric, and/or speech-language delays/disorders. Furthermore, all participants passed the screening Checklist for Auditory Processing in Adults (SCAP-A) [27]. All participants provided informed consent in accordance with the ethical guidelines for bio-behavioral research at the All India Institute of Speech and Hearing, Mysore [28].

### 2.2. Stimuli

We recorded obligatory cortical auditory evoked potentials (CAEPs) in response to bisyllabic (Consonant-Vowel-Consonant-Vowel—CVCV) word stimuli. The target stimuli consisted of four bisyllabic, meaningful, and frequently used Kannada words (commonly encountered in high school textbooks): /ɡaɖi/, /ɡaðe/, /gadʒa/, and /ganja/, with each word beginning with the syllable /ga/. We specifically chose these words to ensure that the primary acoustic variation occurred in the second syllable, requiring participants to pay attention to the entire word before responding. To create the stimuli, we recorded a native female Kannada speaker producing these words, each repeated ten times consecutively, while minimizing inflections and intonations. After recording, we carefully selected the clearest and most acoustically robust utterance for each word by visually inspecting the waveform and spectrogram, and through auditory evaluation. The final stimulus durations were 463.2 ms for /ɡaɖi/, 486.1 ms for /ɡaðe/, 437.5 ms for /gadʒa/, and 580 ms for /ganja/. The stimuli were presented either in a quiet background or in the presence of a speech-shaped noise (SSN). To generate the SSN, we used a custom Matlab (R2016a) script [29] that generated a noise with the same long-term average speech spectrum as that of a 4-talker babble.

### 2.3. Evoked Potential Recording

All ERP measurements were conducted in a quiet, well-lit room with background noise levels meeting the ANSI S3.1 (2013) standards. ERP responses were recorded using a 256-channel Electrical Geodesic Inc. (EGI) system connected to a GES-400 amplifier. Electrode impedance was ensured to be below 50 kΩ at all channels [30]. We presented the stimuli (words and background noise) using the E-Prime 2.0 software. The stimuli were delivered binaurally via ER-3A insert earphones at 70 dB SPL, with an onset-to-onset inter-stimulus interval of 3 s and an onset jitter of 0.3 s. The stimuli were presented in a manner similar to a multiple oddball paradigm [31,32]. Each recording included a total of 100 stimuli, presented in a pseudorandom order. The presentation probability was 7:1:1:1, meaning that the target stimulus /ɡaɖi/ was played 70 times, while the other three stimuli, which acted as ‘distractors’, were played 10 times each. In background masker conditions, the SSN was presented at 60 dB SPL to achieve a signal-to-noise ratio (SNR) of +10 dB. The output levels of both the target stimuli and the background noise were verified at 70 dB SPL and 60 dB SPL (effective SNR = +10 dB), respectively, using a calibrated sound level meter. Calibration was checked before testing each participant to ensure consistent stimulus presentation levels across participants. Further, to prevent cortical responses from being confounded by the noise onset, the masker was initiated five seconds before the first stimulus and continued for five seconds after the last stimulus.

EEG recordings from each participant were collected under four conditions (~330 s per condition), which included two background conditions (quiet, SSN) and two attention conditions (active, passive). EEG recording was performed at a sampling rate of 1000 Hz with open filter settings, using 256 active electrodes and ‘Cz’ as the reference electrode. We always recorded passive attention conditions first to minimize potential bias toward the target words if active conditions were conducted beforehand. For passive attention conditions, we instructed participants to ignore the sounds and watch a muted, closed-captioned movie. In active attention conditions, participants responded by pressing the appropriate buttons for each of the four stimuli while ignoring any background noise when present. Before the start of this condition, all participants were given practice runs to minimize errors in accuracy due to mistakes in button presses. They were also instructed to maintain visual focus on a static image that displayed written instructions indicating which button corresponded to each stimulus. Although response accuracy and reaction time for the button press were not included in the final analysis, all participants demonstrated at least 80% accuracy during the active attention condition, ensuring adequate task engagement.

### 2.4. Response Pre-Processing

The raw EEG data were analyzed offline using the EEGLAB package (version 14.1.1) [33]. To optimize data handling, we downsampled the continuous EEG data, recorded at 1000 Hz, to 256 Hz. We then applied a bandpass filter (1–30 Hz) to remove low-frequency drifts and high-frequency noise, visually inspected the filtered data to identify and remove bad channels and segments with large artifacts, typically caused by body movements. We also used the EEGLAB plug-in CleanLine to eliminate the 50 Hz line interference and its harmonic frequencies. Any rejected channels were interpolated using a spherical spline interpolation method, and we re-referenced the data to a Common Average Reference (CAR), calculated as the mean activity across all electrode channels. Next, we performed Independent Component Analysis (ICA) with a Principal Component Analysis (PCA) dimension reduction to 64 components, selecting the components that explained the greatest variance in the data. Additionally, we also excluded components that explained less than 1% of the data variance, as indicated in the component summary plots provided by the EEGLAB platform. Using visual inspection, supplemented by the EELAB plugin ICLabel [34], we identified and removed non-brain responses corresponding to eye blinks, horizontal and vertical ocular movements, heartbeat artifacts, incomplete/inefficient IC decomposition, and muscular and other physiological noise sources.

**Figure 1:**
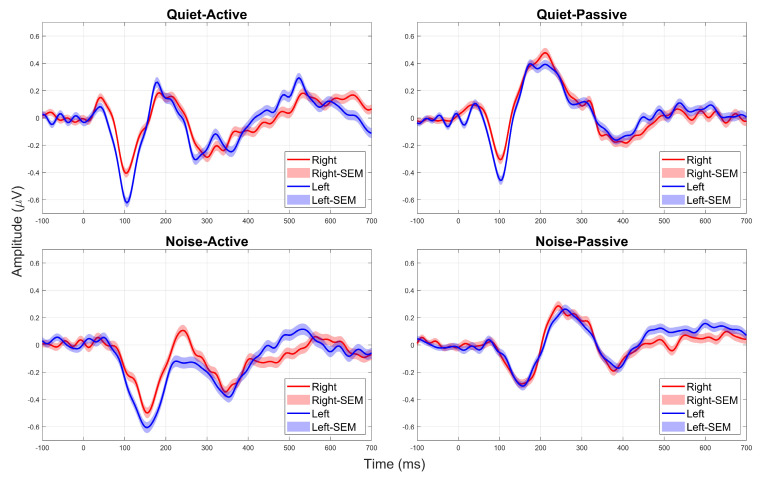
Mean waveforms from right (red tracings) and left (blue tracings) hemispheres across the four different conditions. Lighter shaded areas indicate the standard error of the mean for each condition. The responses measured under quiet backgrounds are on the top, and those measured under the SSN background are at the bottom. The responses measured under the active attention condition are shown on the left, and those measured under passive attention are shown on the right.

Once the data were cleaned, we epoched the data from −200 ms to 1000 ms, considering only responses to the target stimulus (/ɡaɖi/), with stimulus onset at 0 ms. We applied baseline correction using the pre-stimulus period (−200 to 0 ms) and rejected any epochs exceeding ±50 µV to maintain data quality. We then segmented the data by hemisphere, grouping all scalp electrodes on the left and right sides of the midline, resulting in 81 electrodes per hemisphere. We excluded electrodes covering the neck, jaw, and face, as these had a higher likelihood of contamination from non-brain activity. Finally, we averaged the epochs separately for the four conditions in each hemisphere, ensuring that each averaged response was derived from at least 56 clean epochs per condition after pre-processing. Figure 1 shows the grand-averaged responses of the right and left hemispheres across the four conditions.

### 2.5. Data Analysis

As described earlier, we used the bisyllabic word (/ɡaɖi/) as the target stimulus. Since obligatory cortical auditory responses (P1-N1-P2) are sensitive to within-stimulus changes [17,35], we expected to observe two obligatory responses corresponding to the two CV portions of the stimulus. However, prior research indicates that the P1 component is often reduced in amplitude or even absent in young adult listeners [36]. Additionally, we anticipated an overlap between the P2 component for the first CV and the P1 component for the second CV, potentially contaminating the P2 response for the first CV. Due to this overlap, we excluded both P1 and P2 components (as well as combined responses such as P1-N1-P2 or N1-P2) from statistical analyses and focused solely on the N1 component of the first syllable. We also excluded data from the N1 peak of the second syllable, as it was often absent or difficult to identify in several participants, particularly under the passive attention condition and the SSN background condition.

Since the three distractor stimuli were each presented only 10 times per condition, we did not analyze responses to these stimuli. Instead, we identified the peak N1 amplitudes only for the target stimulus (/ɡaɖi/) for all participants across the four conditions. For each of the four conditions, the N1 peak was identified as the most negative voltage point within a pre-defined latency window, adjusting for expected delays due to background noise [37]. The voltage at this time point (in µV) was recorded as the N1 peak amplitude.

## 3. Results

**Figure 2:**
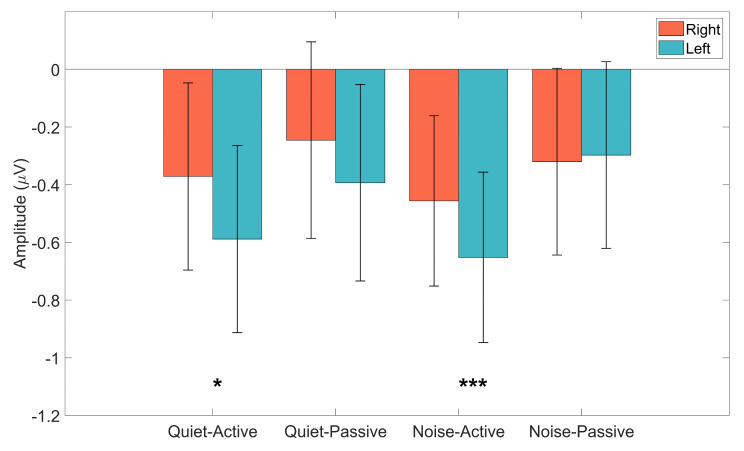
Mean N1 amplitudes for the right (red bars) and left (blue bars) across the four conditions. Vertical error bars indicate ±1 standard deviation. Note: * = *p* < 0.05, *** = *p* < 0.001.

In this study, we examined the interactive effects of background noise (quiet vs. SSN) and attention (active vs. passive) on hemispheric asymmetry in the cortical representation of speech stimuli. Specifically, using high-density EEG recording, we investigated how these factors influence the CAEP N1 peak amplitudes in response to bisyllabic speech stimuli. Figure 1 shows the averaged waveforms across the different conditions, comparing the CAEPs for the right (red color) and left (blue color) hemispheres. From Figure 1, we can see that there is a general trend for the N1 peaks (negative deflections around 100 ms in quiet, and around 160 ms in noise backgrounds) for the left hemisphere to be larger (more negative) than the right for most conditions. Figure 2 shows the mean N1 amplitudes (in µV) across the different conditions. Similar trends from Figure 1 are also observed in Figure 2, with the mean amplitudes of N1 peaks being larger (more negative) for the left hemisphere, in all conditions except the passive attention condition under the noise background.

To quantify these outcomes, N1 peak amplitudes were analyzed using a linear mixed-effects model (LMM) with Hemisphere (Left, Right), Attention (Active, Passive), and Background (Quiet, Noise) entered as fixed effects along with all interaction terms. Participant was included as a random intercept to account for repeated measures and inter-individual variability. Linear mixed-effects modeling was adopted as it allows simultaneous estimation of fixed experimental effects while appropriately modeling within-subject dependence, which is particularly advantageous for electrophysiological data [38].

An ANOVA of the fitted model (Type III sums of squares, Satterthwaite approximation) revealed significant main effects of Hemisphere and Attention, but not Background. Table 1 describes the outcomes of the ANOVA model. Specifically, N1 amplitudes were significantly larger (more negative) in the left hemisphere compared to the right [F(1168) = 19.396, *p* < 0.001, ηp^2^ = 0.104], indicating a medium-to-large hemispheric effect. A significant main effect of Attention was also observed [F(1168) = 28.501, *p* < 0.001, ηp^2^ = 0.145], reflecting robust enhancement of N1 amplitude during active relative to passive listening. The main effect of Background was not significant [F(1168) = 0.017, *p* = 0.897, ηp^2^ < 0.001]. Importantly, a significant Hemisphere × Attention interaction was observed [F(1168) = 6.056, *p* = 0.015, ηp^2^ = 0.035], indicating that hemispheric asymmetry in N1 amplitude varied as a function of attentional state. The Hemisphere × Background interaction showed a trend toward significance (*p* = 0.071), whereas the Background × Attention and three-way interaction terms were not significant (*p* > 0.05).

To characterize the Hemisphere X Attention interaction, estimated marginal means were computed, and planned contrasts were conducted to compare left and right hemisphere N1 amplitudes within each Attention X Background condition. Bonferroni correction was applied to control for multiple comparisons. During active attention, significant hemispheric asymmetry was observed in both background conditions. In the quiet–active condition, N1 amplitudes were significantly larger in the left than the right hemisphere (Δ = −0.318 µV, 95% CI [−0.470, −0.166], *p* < 0.001). A similar pattern was observed in the noise–active condition (Δ = −0.216 µV, 95% CI [−0.368, −0.063], *p* = 0.022). These effects indicate moderate and reliable hemispheric differences during active auditory attention. In contrast, no significant hemispheric differences were observed during passive listening. Neither the noise–passive condition (Δ = 0.015 µV, 95% CI [−0.138, 0.167], *p* = 1.000) nor the quiet–passive condition (Δ = −0.166 µV, 95% CI [−0.318, −0.013], *p* = 0.132) showed reliable left-right differences. Outcomes from the planned contrasts are summarized in Table 2. Taken together, these findings indicate that hemispheric asymmetry of the CAEP N1 is robust during active listening but reduced or absent during passive listening, irrespective of background noise.

## 4. Discussion

This study investigated how attention and background noise interact to shape hemispheric asymmetry in cortical auditory processing, as indexed by N1 amplitudes of the cortical auditory evoked potential. Linear mixed-effects analyses revealed robust main effects of Hemisphere and Attention, with N1 amplitudes being significantly larger (more negative) in the left compared to the right hemisphere and during active compared to passive listening. In contrast, background noise did not exert a significant main effect on N1 amplitude, nor did it interact with attention or hemisphere. Importantly, a significant Hemisphere × Attention interaction indicated that hemispheric asymmetry depended on attentional state. Follow-up comparisons demonstrated clear left-hemisphere dominance during active listening in both quiet and noise conditions, whereas no significant hemispheric differences were observed during passive listening, irrespective of background. Thus, active attention was associated with enhanced left-hemisphere engagement, while passive listening resulted in more bilaterally balanced cortical responses. Together, these findings suggest that attentional state, rather than background noise, plays a critical role in modulating hemispheric asymmetry in early cortical auditory processing.

The effects of attention [14,39,40] and background noise [18,41,42] on CAEPs are well documented. Attention has consistently been shown to enhance CAEP amplitudes, often with minimal effects on response latency, whereas background noise has been reported to reduce amplitudes and delay responses depending on task demands and stimulus characteristics. Although the present study was not designed to isolate these general effects, our findings were broadly consistent with prior work. N1 amplitudes were significantly larger during active attention compared to passive attention conditions across both background conditions, while background noise did not result in statistically significant changes in N1 peak amplitudes.

The significant main effect of Hemisphere, reflecting overall larger N1 amplitudes in the left compared to the right hemisphere, indicates a general left-hemisphere dominance in cortical processing of speech stimuli. While previous studies have reported variability in hemispheric asymmetry [39,43], the present findings align with a substantial body of literature demonstrating left-lateralized processing of speech [10,11,20,44]. The left hemisphere is widely considered the primary neural substrate for speech perception and production [45]. Given that the stimuli in the current study consisted of meaningful bisyllabic words, preservation of left-hemisphere dominance across conditions is expected. However, the reduced hemispheric asymmetry observed during passive listening suggests that diminished cognitive engagement may attenuate left-hemisphere specialization, resulting in more bilaterally distributed cortical processing.

A central finding of this study was that attention played a more dominant role than background noise in modulating hemispheric asymmetry in cortical auditory processing. Our results showed a clear left hemispheric dominance for active attention, while passive attention revealed more bilateral processing of speech stimuli, irrespective of them being presented in quiet or in the presence of SSN. Importantly, background noise did not significantly modulate hemispheric asymmetry, indicating that attentional state, rather than acoustic context, was the primary driver of lateralized cortical processing.

The role of attention on hemispheric asymmetry is still underexplored, and the existing literature is divided in its outcomes. Several studies have suggested that active attention results in right hemisphere dominance using fMRI [21,22] as well as CAEPs [23,46]. On the other hand, Poeppel et al. (1996) [24] demonstrated stronger activity over the left hemisphere for an active discrimination task, while there were minimal differences between the two hemispheres under passive attention. In their study, Zhang et al. (2014) [39] also did not see an obvious difference between right and left hemispheres for the N1-P2 complex measured under passive attention conditions. The outcomes from our study are more closely aligned with Poeppel et al. (1996) [24], likely because of the similarities in the stimuli being used. While studies reporting right-hemisphere dominance under active attention have typically employed simple tonal stimuli, our study, as well as Poeppel et al. (1996) [24], used speech stimuli. Furthermore, while Poeppel et al. (1996) [24] used monosyllabic non-meaningful speech stimuli, our study took this a step further by using meaningful bisyllabic words. Taken together, these findings suggest that attentional modulation of hemispheric asymmetry is strongly influenced by stimulus type, with speech stimuli preferentially engaging left-hemisphere mechanisms during active listening.

The effect of background noise on hemispheric asymmetry appears to be relatively consistent across studies. Although some studies have reported enhanced left-hemispheric dominance in noisy conditions [47], the general trend is for a rightward shift in the hemispheric activity in the presence of background noise [19,20]. Specifically, Shtyrov and colleagues found that the mismatch negativity (MMNm) response, primarily generated in the left hemisphere in quiet conditions, exhibited significantly reduced left-hemispheric dominance under noisy conditions, indicating a redistribution of cortical activity. In contrast to these findings, background noise did not significantly modulate hemispheric asymmetry in the present study. Neither a main effect of background noise nor interactions involving background noise were observed, indicating that left-right differences in N1 amplitude were preserved across quiet and noise conditions. This discrepancy may reflect differences in task demands, attentional engagement, and stimulus type across studies. Whereas prior work often employed passive listening paradigms and non-speech or deviant-detection tasks (e.g., MMN), the present study required explicit attention to meaningful speech stimuli in the active condition, which may have stabilized left-hemisphere dominance even under acoustically degraded conditions. Future studies employing more linguistically complex informational maskers, such as multi-talker speech babble, may further elucidate how background noise interacts with attentional state to influence hemispheric asymmetry during speech processing.

This dynamic modulation of hemispheric specialization by attentional state across acoustic contexts reinforces the idea that auditory processing is not strictly hardwired but rather adaptable based on cognitive demands. The preservation of left-hemisphere dominance during active listening in noisy environments may have important implications for speech perception in everyday communication settings. In real-world scenarios, listeners often encounter background noise (e.g., in restaurants, classrooms, and workplaces), which requires selective attention mechanisms to extract relevant speech cues. The present findings suggest that active attention plays a crucial role in maintaining efficient speech processing under these conditions. Notably, individuals who naturally exhibit greater left-hemisphere engagement may be better equipped to sustain effective speech perception when faced with challenging auditory environments.

The study by Slugocki et al. (2022) [11] provides further support for this interpretation by demonstrating that listeners with stronger left-hemisphere lateralization for speech-related CAEPs also perform better on speech-in-noise tasks. Their study, conducted in older adults with hearing loss, found that leftward lateralization of the N1-P2 complex was a significant predictor of speech-in-noise performance, independent of hearing aid processing and audiometric thresholds. This suggests that individual differences in cortical lateralization may contribute to variability in speech perception abilities. Future studies with larger sample sizes could investigate whether similar patterns extend to other ERP components, such as the P300 or MMN, as well as to measures of cortical entrainment using more ecologically valid continuous speech stimuli [48]. Additionally, examining whether attentional training or neuroplasticity-based interventions can enhance left-hemisphere engagement and improve speech-in-noise perception may have important clinical implications.

A possible limitation of the present study is that, although the task was designed to emphasize auditory perception, active listening necessarily engages additional task-related processes, including visual monitoring and motor preparation. While such processes can influence cortical activity at later latencies, the present analyses focused on the early N1 time window, which precedes overt motor responses and is widely considered an index of early auditory cortical processing modulated by attention. Future studies incorporating balanced cross-modal control tasks or explicit modeling of behavioral covariates may further refine the specificity of attentional contributions to hemispheric asymmetry.

Additionally, although latency measures can provide complementary insight into network-level recruitment and sensory gating mechanisms, the present study focused specifically on N1 amplitude as an index of hemispheric engagement. Latency analyses were not pursued because the experimental design and preprocessing pipeline were optimized for amplitude-based comparisons rather than precise estimation of peak timing across conditions. Future studies explicitly designed to examine latency shifts, particularly under varying attentional and masking demands, may further elucidate the contribution of thalamo-cortical and association-level networks to speech processing.

## 5. Conclusions

This study demonstrates that attention plays a more dominant role than background noise in modulating hemispheric asymmetry in cortical auditory processing, with active engagement reinforcing left-hemisphere dominance for speech processing. In contrast, passive listening, irrespective of background noise, was associated with a more balanced hemispheric response, suggesting a shift toward bilateral auditory processing when cognitive resources are not actively allocated. These findings highlight that auditory speech processing is not static but dynamically shaped by cognitive engagement across different acoustic contexts, with important implications for speech perception in noise, auditory rehabilitation, and clinical interventions.

Future research should explore how linguistically relevant background noise, such as informational maskers (e.g., multi-talker babble or competing speech), modulates hemispheric representation, as these conditions introduce additional cognitive and linguistic interference beyond energetic masking. Additionally, the impact of aging, bilingualism, and hearing loss on attentional modulation of hemispheric asymmetry warrants further investigation. By expanding research to include linguistically complex noise conditions and diverse populations, future work can refine our understanding of how cognitive and auditory mechanisms interact to shape speech processing in real-world scenarios, ultimately leading to more effective interventions for individuals struggling with speech-in-noise perception.

## Figures and Tables

**Table 1:** Linear mixed-effects model results for N1 amplitude.

Effect	df	F	*p*	ηp^2^
Hemisphere	1168	19.396	<0.001	0.104
Background	1168	0.017	0.897	<0.04
Attention	1168	28.501	<001	0.145
Hemisphere × Background	1168	3.311	0.071	0.019
Hemisphere × Attention	1168	6.056	0.015	0.035
Background × Attention	1168	0.762	0.384	0.005
Hemisphere × Background × Attention	1168	0.251	0.617	0.001

Note. Type III sums of squares; Satterthwaite degrees of freedom. ηp^2^ = partial eta squared. Shaded areas signify statistically significant outcomes.

**Table 2:** Planned hemisphere contrasts within each condition.

Condition	Δ (Left − Right, µV)	95% CI	*p* (Bonferroni)
Quiet–Active	−0.318	[−0.470, −0.166]	<0.001
Noise–Active	−0.216	[−0.368, −0.063]	0.022
Quiet–Passive	−0.166	[−0.318, −0.013]	0.132
Noise–Passive	0.015	[−0.138, 0.167]	1.000

Note. Negative values indicate larger (more negative) N1 amplitudes in the left hemisphere. Shaded areas signify statistically significant outcomes (after Bonferroni’s corrections are applied.

## Data Availability

The original contributions presented in this study are included in the article. Further inquiries can be directed to the corresponding author.

## Abbreviations

The following abbreviations are used in this manuscript:

CAEPs	Cortical Auditory Evoked Potentials
EEG	Electroencephalography
ERPs	Event-Related Potentials
N1–P2	N1–P2 complex
MMN	Mismatch Negativity
MMNm	Magnetic Mismatch Negativity
MEG	Magnetoencephalography
fMRI	Functional Magnetic Resonance Imaging
SSN	Speech-Shaped Noise
SNR	Signal-to-Noise Ratio
SPL	Sound Pressure Level
CVCV	Consonant–Vowel–Consonant–Vowel
LMM	Linear Mixed-Effects Model
ICA	Independent Component Analysis
PCA	Principal Component Analysis
CAR	Common Average Reference
EEGLAB	MATLAB-based EEG analysis toolbox
EGI	Electrical Geodesic Inc.
GES	Geodesic EEG System
ANSI	American National Standards Institute
SCAP-A	Screening Checklist for Auditory Processing in Adults
